# Chronic Kidney Disease and Diabetic Retinopathy in Patients with Type 2 Diabetes

**DOI:** 10.1371/journal.pone.0149448

**Published:** 2016-02-17

**Authors:** Antonio Rodríguez-Poncelas, Xavier Mundet-Tudurí, Sonia Miravet-Jiménez, Aina Casellas, Joan F. Barrot-De la Puente, Josep Franch-Nadal, Gabriel Coll-de Tuero

**Affiliations:** 1 Primary Health Care Center Anglès, Gerència Territorial Girona, Institut Català de la Salut, Girona, Spain; 2 Unitat de Suport a la Recerca Girona, Institut Universitari d’Investigació en Atenció Primària Jordi Gol (IDIAP Jordi Gol), Barcelona, Spain; 3 Unitat de Suport a la Recerca Barcelona Ciutat, Institut Universitari d'Investigació en Atenció Primària Jordi Gol (IDIAP Jordi Gol), Barcelona, Spain; 4 Universitat Autonoma de Barcelona, Bellaterra, Spain; 5 Primary Health Care Center Martorell, Gerència Territorial Metropolitana Sud, Institut Català de la Salut, l’Hospitalet de LLobregat, Barcelona, Spain; 6 Primary Health Care Center Jordi Nadal (Salt), Gerència Territorial Girona, Institut Català de la Salut, Girona, Spain; 7 Primary Health Care Center Raval Sud, Gerència d’Àmbit d’Atenció Primària Barcelona Ciutat, Institut Català de la Salut, Barcelona, Spain; 8 CIBER of Diabetes and Associated Metabolic Diseases (CIBERDEM), Instituto de Salud Carlos III (ISCIII), Madrid, Spain; Innsbruck Medical University, AUSTRIA

## Abstract

**Purpose:**

To explore the relationship between chronic kidney disease (CKD) and diabetic retinopathy (DR) in a representative population of type 2 diabetes mellitus (DM2) patients in Catalonia (Spain).

**Methods:**

This was a population-based, cross-sectional study. A total of 28,344 patients diagnosed with DM2 who had recorded ophthalmologic and renal functional examinations were evaluated. Data were obtained from a primary healthcare electronic database of medical records. CKD was defined as an estimated glomerular filtration ratio (eGFR) of <60 ml/min/1.73m^2^ and/or urine albumin to creatinine ratio (UACR) ≥30 mg/g. DR was categorized as non-vision threatening diabetic retinopathy and vision threatening diabetic retinopathy.

**Results:**

CKD was associated with a higher rate of DR [OR], 95% confidence interval [CI], 1.5 (1.4–1.7). When we analyzed the association between different levels of UACR and DR prevalence observed that DR prevalence rose with the increase of UACR levels, and this association was significant from UACR values ≥10 mg/g, and increased considerably with UACR values ≥300mg/g (Odds ratio [OR], 95% confidence interval [CI], 2.0 (1.6–2.5). This association was lower in patients with eGFR levels 44 to 30 mL/min/1.73m^2^ [OR], 95% confidence interval [CI], 1.3 (1.1–1.6).

**Conclusions:**

These results show that CKD, high UACR and/or low eGFR, appear to be associated with DR in this DM2 population.

## Introduction

Chronic kidney disease (CKD) and diabetes retinopathy (DR) are two major microvascular complications found in long-standing type 1 (DM1) and type 2 diabetic (DM2) patients. In addition to sharing risk factors, such as poor glycaemia control and systolic hypertension, CKD and DR are reflected in the clinical manifestations of similar microvascular lesions in the glomerular and retinal vessels [1–3]. UACR and eGFR are clinically markers for evaluate renal function.

Urine albumin to creatinine ratio (UACR) and low estimated glomerular filtration rate (eGFR) are clinical markers of renal function. CKD is often associated with DR in DM1 [4, 5], and in some studies in DM2 [6–8]. However, this relationship has not been properly investigated in DM2, and there are few data on which of the two markers of CKD, eGFR or UACR, is more closely related to DR.

High UACR is a marker of endothelial dysfunction and may influence the microvasculature of the kidney and retina. However some studies [9–10] found a relationship between higher UACR levels and DR in DM1 patients but not in DM2 patients. Sabanayagam et al. [11] observed that CKD was associated with DR only in the presence of albuminuria, suggesting that CKD is more likely related to diabetes in the presence of albuminuria. Previous studies have shown that UACR not only is an important clinical marker for CKD, but also is closely associated with the progression of DR [12]. Moreover, the relationship between eGFR with DR remains unclear, particularly in DM2. Man et al. [13] observed that lower levels of eGFR were associated with the presence and severity of DR. A recent report showed that both an elevated UACR and decreased eGFR predicted the development of DR in DM2 patients with the former having a greater impact [14].

The aim of this study was to investigate the association between CKD, UACR and/or eGFR, and DR in a representative Catalonian (Spain) DM2 population.

## Materials and Methods

A population-based, cross-sectional study was performed in Catalonia (Spain) with patients aged between 30 and 90 years (at 31st December, 2012), diagnosed with DM2 prior to retinal photography screening, and whose DR category was recorded in their medical records. The most recent retinal photography registered was selected and employed as the index date. The criteria for the diagnosis of type DM2 were those established by the American Diabetes Association [15] at the time of registration in the electronic database.

Data were obtained from the SIDIAP (System for Research and Development in Primary Care) electronic database. The SIDIAP includes data from the primary healthcare electronic medical records (e-CAP) on demographic information, appointment dates with doctors and nurses, clinical diagnoses, clinical variables, prescriptions written, referrals to specialists and hospitals, results from laboratory tests, and medication sold by pharmacies. The quality of the SIDIAP data has been previously documented, and the database has been widely used to study the epidemiology of a number of health outcomes [16, 17].

### Measures of Kidney Diabetic Disease

Serum creatinine levels and UACR were determined. CKD was defined as UACR >30 mg/g and/or eGFR <60 mL/min/1.73m^2^.

Normoalbuminuria was defined as UACR <30 mg/g; microalbuminuria as UACR 30–299 mg/g; and macroalbuminuria as UACR ≥300 mg/g. The Chronic Kidney Disease Epidemiology Collaboration equation was employed to measure eGFR [18].

### Assessment of Retinopathy

In a large, community-based screening programmer, such as that employed in our study, fundus photography is considered to be the preferred option [19]. Photographs are captured from each eye and the severity of DR is categorized according to the international clinical diabetic retinopathy severity scales recommended by the Global Diabetic Retinopathy Project Group [20] as: non-vision threatening DR (non-VTDR) and vision threatening DR (VTDR). The former includes no apparent retinopathy (no DR); mild non-proliferative DR (mild NPDR); and moderate non-proliferative DR (moderate NPDR). The latter is composed of severe non-proliferative DR (severe NPDR); proliferative DR (PDR); and diabetic macular edema (DME). In our study, retinal photography was performed by skilled personnel using a non-midriatic camera. Subsequently, in the primary health care center, a family physician trained in reading eye fundus photographs registered the result in the patient’s medical records. Each eye was given a DR grade according to the worst result. In the case of patients having more than one fundus photograph during this period, the most recent was employed for analysis. In the study we only included retinal photographs from patients undergoing diabetic retinopathy screening performed at primary health care centers.

### Clinical variables

The following data were obtained from each patient: age, age at diagnosis of diabetes, duration of diabetes, gender, and glycated hemoglobin levels (A1C). Cardiovascular risk factors including body mass index (BMI), total cholesterol, low-density (LDL) cholesterol, high density lipoprotein (HDL) cholesterol, non-HDL cholesterol, systolic and diastolic blood pressure (SBP and DBP), pulse pressure (PP), and smoking status according to the last condition registered before the index date were collected. Data for clinical variables were gathered from the 15 months prior to the index date with the exception of blood pressure, PP, and BMI, which were obtained from the previous 12 months. Additional data were gathered on medication.

The study was approved by the Ethics Committee of the Primary Health Care University Research Institute Jordi Gol (protocol number 13/137). All patient records and information was anonymized and de-identified prior to analysis.

### Statistical analysis

A descriptive analysis stratified by renal function (according to eGFR and UACR) and retinography result was performed. The absolute and relative frequencies of the qualitative variables, and their mean and standard deviation (or median and interquartile range according to the characteristics of the variable) were calculated.

In order to study whether the clinical variables of interest depended on renal function, Pearson’s chi square test and analysis of variance (ANOVA) were employed for means and proportions, respectively. The hypothesis contrast was bilateral in all cases with a 0.05 level of significance.

In addition, the relationship between renal function values (according to eGFR and UACR) and the presence of retinopathy was assessed by logistic regression models adjusted by age, sex, age at retinography, duration of DM2, systolic blood pressure, A1C ≤7%, smoking, BMI, and cardiovascular disease.

Analyses were performed with Stata/SE version 13 for Windows (Stata Corp., College Station, Texas, USA).

## Results

Prior to retinography 28,344 DM2 patients had their eGFR and UACR values registered. From the total sample, 14.6% had an eGFR <60 mL/min/1.73m^2^, women had lower rates than men (p<0.001). As can be observed in **Table 1**, diabetes duration, hypertension, cardiovascular disease (CVD), and treatment with insulin displayed a direct association with decreased eGFR.

**Table 1:** Patients’ characteristics with respect to glomerular filtration rate (eGFR).

Variable	Global	eGFR ≥60	eGFR 59–45	eGFR 44–30	eGFR <30	p-value^a^
**Female, n (%)**	12,520 (44.2)	10,363 (42.8)	1,478(51.2)	568 (53.9)	111 (52.6)	<0.001
**Hypertension, n (%)**	22,971 (81.0)	19,001 (78.5)	2,737 (94.8)	1,026 (97.4)	207 (98.1)	<0.001
**Any cardiovascular disease**^b^**, n (%)**	3,969 (14.0)	3,026 (12.5)	590 (20.4)	286 (27.2)	67 (31.8)	<0.001
**Coronary Heart disease, n (%)**	3,048 (10.8)	2,326 (9.6)	446 (15.5)	221 (21.0)	55 (26.1)	<0.001
**Stroke, n (%)**	1,148 (4.1)	854 (3.5)	186 (6.4)	90 (8.5)	18 (8.5)	<0.001
**Insulin treatment, n (%)**	4,737 (16.7)	3,781 (15,6)	567 (19.6)	302 (28.7)	87 (41.2)	<0.001
**Age T2DM (years), mean (SD)**	58.8(10.5)	57.5(10.2)	65.6 (9.0)	67.2 (9.7)	65.0(10.1)	<0.001
**Age at retinography (years), mean (SD)**	65.7(10.7)	64.3(10.4)	73.7(7.9)	76.1 (7.8)	74.6(8.9)	<0.001
**Diabetes duration (years), mean (SD)**	7.0 (5.2)	6.8 (5.0)	8.2 (5.6)	9.0 (6.0)	9.7(6.1)	<0.001
**A1C (%), mean (SD)**	7.4 (1.4)	7.4(1.4)	7.2 (1.2)	7.3 (1.4)	7.2 (1.3)	<0.001
**Non-HDL cholesterol (mg/dL), mean (SD)**	137.3(35.2)	137.8(35.2)	135.4(34.6)	132.5(35.2)	132.5(38.2)	<0.001
**Hemoglobin (g/dL), mean (SD)**	14.0 (1.5)	14.1 (1.4)	13.3 (1.6)	12.7 (1.6)	12.0 (1.5)	<0.001
**SBP (mmHg), mean (SD)**	134.9(12.6)	134.8(12.5)	136.1(13.1)	135.8(13.5)	133.9(14.0)	<0.001
**DBP (mmHg), mean (SD)**	76.4 (8.3)	76.9 (8.2)	74.2 (8.2)	71.9 (8.3)	70.4 (8.3)	<0.001
**Heart Rate (bpm), mean (SD)**	76.1 (12.1)	76.3 (12.0)	75 (12.5)	74.2 (12.5)	72.5 (12.0)	<0.001

^a^P value for comparison of groups by glomerular filtration rate with Pearson’s chi-square test for qualitative variables and t-test for quantitative ones.

^b^Any cardiovascular disease includes coronary heart disease and/or stroke

A1C, glycated hemoglobin; bpm, beats per minute; DBP, Diastolic Blood Pressure; DR: diabetic retinopathy.

In contrast, an inverse relationship between eGFR and non-HDL cholesterol, plasma hemoglobin, heart rate, and DBP was reported. With respect to UACR values (**Table 2**), 16.0% were ≥30 mg/g with higher levels being found in men (p<0.001). DM2 duration, poor glycemic control (glycated hemoglobin; A1C >7%), SBP, increased heart rate, hypertension, CVD, and treatment with insulin showed a direct relationship with an increase in UACR levels. An inverse relationship, however, was observed between UACR and plasma hemoglobin levels.

**Table 2:** Patients’ characteristics with respect to urine albumin to creatinine ratio (UACR).

Variable	Global	UACR <30 mg/g	UACR = 30–299 mg/g	UACR ≥300 mg/g	p-value^a^
**Female, n (%)**	12,520 (44.2)	10,886 (45.7)	1,443 (36.4)	191 (33.7)	<0.001
**Hypertension, n (%)**	22,971 (81.0)	18,857 (79.2)	3,573 (90.2)	541 (95.4)	<0.001
**Any cardiovascular disease**^**b**^**, n (%)**	3,969 (14.0)	2,996 (12.6)	830 (21.0)	143 (25.2)	<0.001
**Coronary Heart disease, n (%)**	3,048 (10.8)	2,321 (9.7)	616 (15.6)	111 (19.6)	<0.001
**Stroke, n (%)**	1,148 (4.1)	842 (3.5)	268 (6.8)	38 (6.7)	<0.001
**Insulin treatment, n (%)**	4,737 (20.0)	3,512 (17,9)	1,028 (29.0)	197 (39.4)	<0.001
**Age T2DM (years), mean (SD)**	58.8(10.5)	58.6(10.4)	59.8(10.9)	59.1(10.5)	<0.001
**Age at retinography (years), mean (SD)**	65.7(10.7)	65.4(10.6)	67.6(11.0)	67.6(11.0)	<0.001
**Diabetes duration (years), mean (SD)**	7.0 (5.2)	6.8 (5.1)	7.8 (5.4)	8.6 (5.8)	<0.001
**A1C (%), mean (SD)**	7.4 (1.4)	7.3(1.3)	7.7 (1.6)	7.8 (1.7)	<0.001
**Non-HDL cholesterol (mg/dL), mean (SD)**	137.3(35.2)	137.4 (34.7)	136.5(37.6)	136.6(37.3)	0.320
**Hemoglobin (g/dL), mean (SD)**	14.0 (1.5)	14.0 (1.5)	13.8 (1.7)	13.5 (1.9)	<0.001
**SBP (mmHg), mean (SD)**	134.9(12.6)	134.2(12.2)	138.3(13.7)	141.8(15.8)	<0.001
**DBP (mmHg), mean (SD)**	76.4 (8.3)	76.4 (8.1)	76.4 (9.0)	76.7 (10.0)	0.736
**Heart Rate (bpm), mean (SD)**	76.1 (12.1)	76.0(12.0)	76.6 (12.8)	77.5 (13.2)	0.002

^a^P value for comparison of groups by urine albumin to creatinine ratio (ACR) with Pearson’s chi-square test for qualitative variables and t-test for the quantitative ones.

^b^Any cardiovascular disease includes coronary heart disease and/or stroke

A1C, glycated hemoglobin; DBP, diastolic blood pressure; SBP, systolic blood pressure.

In **Table 3** the characteristics of the patients with respect to the presence or absence of DR can be observed. Duration of diabetes, A1C levels, SBP, heart rate, CVD, and insulin treatment all displayed a direct relationship with DR. An inverse relationship, however, was observed for DR with non-HDL cholesterol, plasma hemoglobin, and DBP.

**Table 3:** Patients’ characteristics with respect to diabetic retinopathy.

Variable	Global	Non DR	Non-VTDR	VTDR	p-value^a^
**Female, n (%)**	12,520 (44.2)	11,089 (44.3)	1,316 (43.8)	115 (40.2)	0.345
**Hypertension, n (%)**	22,971 (81.0)	20,141 (80.4)	2,589 (86.1)	241 (84.3)	<0.001
**Any cardiovascular disease,** ^**b**^ **n (%)**	3,969 (14.0)	3,347 (13.4)	553 (18.4)	69 (24.1)	<0.001
**Coronary heart disease, n (%)**	3,048 (10.8)	2,602 (10.4)	396 (13.2)	50 (17.5)	<0.001
**Stroke, n (%)**	1,148 (4.1)	935 (3.7)	187 (6.2)	26 (9.1)	<0.001
**Insulin treatment, n (%)**	4,737 (20.0)	3,529 (17.1)	1,067 (38.8)	141 (52.8)	<0.001
**Age DM2 (years), mean (SD)**	58.8(10.5)	58.8 (10.4)	58.1 (11.2)	58.4 (11.6)	<0.001
**Age at retinography (years), mean (SD)**	65.7(10.7)	65.6 (10.6)	67.1 (10.8)	67.9 (10.9)	<0.001
**Diabetes duration (years), mean (SD)**	7.0 (5.2)	6.7 (5.0)	9.0 (5.8)	9.5 (5.8)	<0.001
**A1C (%), mean (SD)**	7.4 (1.4)	7.3 (1.3)	7.9 (1.6)	8.2 (1.7)	<0.001
**Non-HDL cholesterol (mg/dL), mean (SD)**	137.3(35.2)	137.8 (35.0)	133.0 (35.5)	133.9 (40.1)	<0.001
**Hemoglobin (g/dL), mean (SD)**	14.0 (1.5)	14.0 (1.5)	13.7 (1.6)	13.7 (1.8)	<0.001
**SBP (mmHg), mean (SD)**	134.9(12.6)	134.6 (12.5)	137.4 (13.6)	138.2 (12.7)	<0.001
**DBP (mmHg), mean (SD)**	76.4 (8.3)	76.5 (8.3)	75.7 (8.5)	75.0 (8.3)	<0.001
**Heart Rate (bpm), mean (SD)**	76.1 (12.1)	76.0 (12.1)	76.9 (12.1)	78.2 (12.3)	<0.001

^a^ P value for comparison of groups by glomerular filtration rate with Pearson’s chi-square test for qualitative variables and t-test for the quantitative ones.

^b^ Any cardiovascular disease includes coronary heart disease and/or stroke

A1C, glycated hemoglobin; DBP, diastolic blood pressure; DR: diabetic retinopathy; SBP, systolic blood pressure; VTDR, Vision Threatening Diabetic Retinopathy.

**Fig 1:**
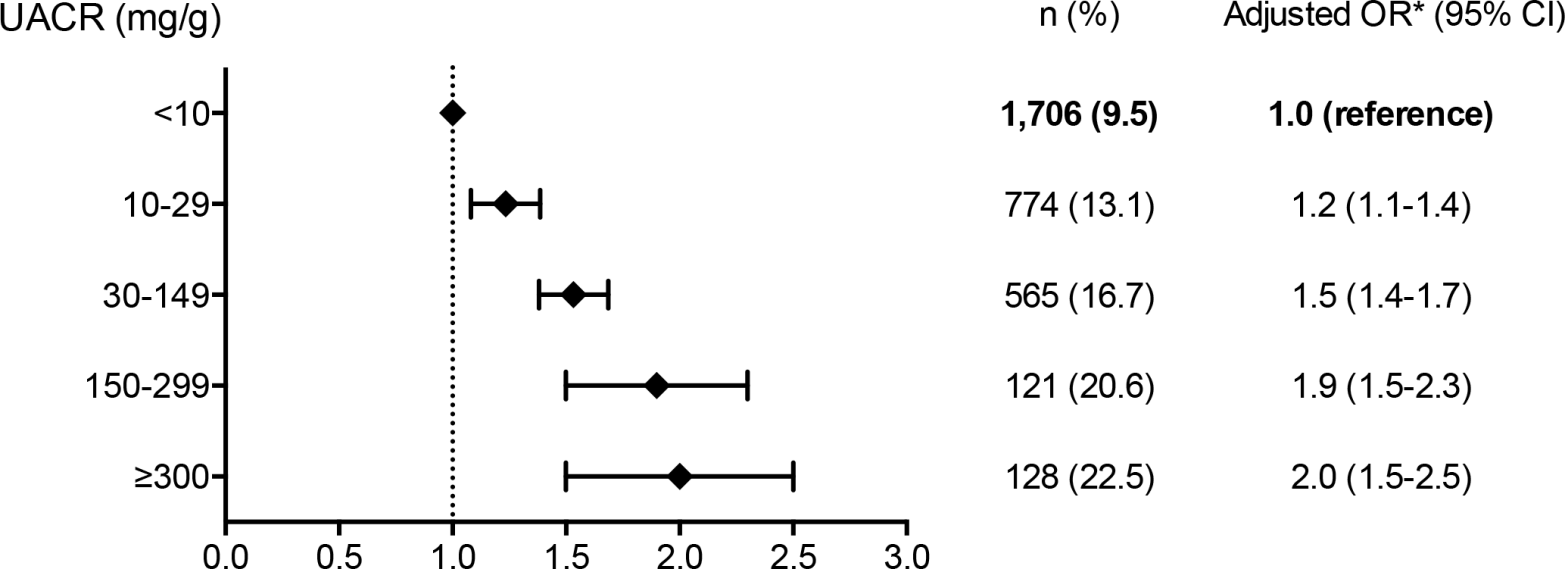
Urine albumin to creatinine ratio and prevalence of diabetic retinopathy. *Adjusted by age, sex, age at retinography, duration type 2 diabetes, systolic blood pressure <140 mmHg, A1C ≤ 7%, smoking, cardiovascular disease, BMI, and eGFR. DR, diabetic retinopathy; OR, odds ratio; UACR, urine albumin to creatinine ratio.

**Table 4** shows that CKD was associated with a higher rate of DR (Odds ratio [OR], 1.5; 95% confidence interval [CI], 1.4–1.7). Patients with elevated UACR had higher rates of DR, particularly if UACR values were ≥300 mg/g (OR, 2.0; 95% CI, 1.6–2.5); this rate was lower in patients with eGFR levels 44 to 30 mL/min/1.73m^2^ (OR, 1.3; 95% CI, 1.1–1.6). Patients with UACR values 30 to 299 mg/gr had DR rate slightly higher than those with eGFR values of 30 to 44 mL/min/1.73m^2^ (OR, 1.5; 95% CI, 1.4–1.7, and OR, 1.3; 95% CI 1.1–1.6, respectively). When we analyzed the association between different levels of UACR and DR prevalence **(Fig 1)** we observed that DR prevalence rose with the increase of UACR levels, and this association was significant from UACR values 10 to 29 mg/g, (OR, 1.2; CI, 1.1–1.4), and increased considerably with UACR values ≥300 mg/g (OR, 2.0; 95% CI, 1.5–2.5).

**Table 4:** Types of chronic kidney disease and diabetic retinopathy.

	OR	(95% CI)	p-value
**UACR (mg/g)**^**a**^			
<30	1.0		
30 to 299	1.5	(1.4, 1.7)	<0.001
≥300	2.0	(1.6, 2.5)	<0.001
**eGFR (mL/min / 1.73m**^**2**^**)**^**a**^			
≥90	1.0		
[60, 89]	1.0	(0.9, 1.1)	0.674
[45, 59]	1.2	(1.0, 1.3)	0.142
[30, 44]	1.3	(1.1, 1.6)	0.009
<30	1.3	(0.9, 1.9)	0.193

^a^Adjusted by age, sex, age at retinography, duration type 2 diabetes, systolic blood pressure <140 mmHg, A1C ≤ 7%, smoking, cardiovascular disease, BMI, UACR and/or eGFR.

Model Diabetic Retinopathy (DR): without DR vs. any type DR (n = 26,897 patients)

DR, diabetic retinopathy; eGFR, estimated glomerular filtration rate; OR, odds ratio; UACR, urine albumin to creatinine ratio.

**Fig 2:**
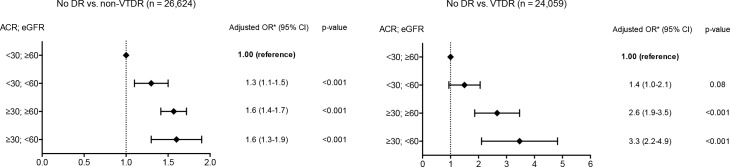
Association of chronic kidney disease and diabetic retinopathy. *Adjusted by sex, age at digital photography, duration of type 2 diabetes, systolic blood pressure <140 mmHg, and A1C ≤7%; DR, diabetic retinopathy; eGFR, estimated glomerular filtration rate; UACR, urine albumin to creatinine ratio; VTDR, vision threatening diabetic retinopathy (severe non proliferative DR, proliferative DR, and diabetic macular edema); non-VTDR, mild non-proliferative DR and moderate non-proliferative DR.

The prevalence of DR using the KDIGO combination of UACR and eGFR categories is shown in **S1 Table**. Moreover, **Fig 2** shows the association between CKD and DR severity. Patients with eGFR <60 mL/min/1.73m^2^ and UACR <30 mg/g had an increased non-VTDR rate (OR 1.3; 95% CI 1.1–1.5) and a non-significant increase in VTDR (OR 1.4; 95% CI 1.0–2.1; p = 0.082). In individuals with UACR ≥30 mg/g and eGFR ≥60 mL/min/1.73m^2^ the rate of VTDR (OR 2.6; 95% CI 1.9–3.5) was higher than non-VTDR (OR 1.6; 95% CI 1.4–1.7).

## Discussion

Our study showed that high UACR level significantly increases the prevalence of diabetic retinopathy in type 2 diabetic patients, even after adjustment for variables, and this association was significant from UACR values ≥10 mg/g. These findings concur with the RIACE study cohort [21]. We observed that individuals with elevated UACR had a higher DR prevalence than those with decreased eGFR. A number of factors could have contributed to the renal and retinal tissue injury observed; including A1C levels, hypertension, dyslipidemia, diabetes duration, age at onset of DM2, smoking, and UACR levels [6, 10, 22, 23]. Patients with CKD are more likely to present with DR VTDR [24]. High UACR doubles the possibility of developing DR with respect to normal UACR levels, a risk that increases considerably with macroalbuminuria, even after adjusting for other factors [7, 14, 25]. In addition, an association was observed between low eGFR and greater prevalence of DR. Lu et al. [26] showed that a decrease in eGFR was significantly correlated with DR, after controlling for sex, age, and albuminuria staging. Wu J et al. [27] observed that, after the adjustment of the variables affecting the relationship between eGFR and DR, results of a univariate analysis suggested that eGFR remained significantly associated with DR. In our study we observed that eGFR levels <45 ml/min/1.73m^2^ were associated with higher DR prevalence, but we did not find this association with eGFR levels ≥45/ml/min/1.73 m^2^. This association was not significant with eGFR levels <30 mL/min/1.73m^2^, probably because there were few patients in this group.

Penno et al. [28] reported an association between UACR ≥300 mg/g and DR (OR 2.9; 95% CI, 2.1–4.0) higher than that observed in our work. Such a variation could be due to the different grouping of DR types. Nevertheless, in both studies elevated UACR had a greater association with DR than decreased eGFR.

CKD in DM2 is more heterogeneous than in type 1 diabetes, retinopathy and albuminuria were both absent in 30% of adults with DM2 and chronic renal insufficiency according to the Third National Health and Nutrition Survey [29]. Patients with diabetes are also susceptible to non diabetic renal disease [30]. A decrease in eGFR with normoalbuminuria in such patients sometimes may be linked to the presence of arteriosclerosis.

Recently, Lee et al. [31] showed a direct association between DR and CKD. The presence of CKD identified a group of DM2 patients of being at a greater risk of presenting macro and microvascular complications. Moreover, the presence of CKD and DR was associated with a more rapid reduction in renal function and greater mortality in this group of patients who might benefit from more aggressive treatment.

Our study should be interpreted with consideration of the following limitations. First, this study was a cross-sectional analysis; therefore, the data were recorded from an electronic database and diabetic patients with DR-VTDR, who were attended by an ophthalmologist or endocrinologist, could have been poorly represented. Second, because of the small number of cases in some categories, we could not carry out the final analysis using all KDIGO categories. Despite these limitations, this study used a nationally representative sample of DM2 patients. Moreover, to the best of our knowledge, this is the first large population based study to examine the association between CKD, decreased eGFR and high UACR, and DR among a representative Catalonian, Spain, DM2 population.

## Conclusions

CKD appears to be associated with DR in this DM2 population. Elevated UACR had a greater association with DR prevalence than decreased eGFR, but both were associated with DR in DM2 patients.

## Supporting Information

S1 Table Prevalence of diabetic retinopathy using KDIGO combinations of UACR and eGFR categories. (DOCX)
