# Supplementary material for: Chronic Kidney Disease and Diabetic Retinopathy in Patients with Type 2 Diabetes
Source: PLoS One. 2016 Feb 17;11(2):e0149448. doi: 10.1371/journal.pone.0149448 (PMC4757564; doi:10.1371/journal.pone.0149448)
Supplement: S1 Table — (DOCX) [file pone.0149448.s001.docx]

**S1 Table**. Prevalence of diabetic retinopathy using KDIGO combinations of UACR and eGFR categories

|  | | Frequency | Percentage | Valid percentage | Cumulative  Percentage |
| --- | --- | --- | --- | --- | --- |
| Values, | eGFR ≥60 & UACR<30 | 20.879 | 73,7 | 73,7 | 73,7 |
|  | eGFR ≥60 & UACR (30, 299) | 2.986 | 10.5 | 10.5 | 84.2 |
|  | eGFR ≥60 & UACR ≥300 | 329 | 1.2 | 1.2 | 85.4 |
|  | eGFR (30, 59) & UACR <30 | 2.853 | 10.1 | 10.1 | 95.4 |
|  | eGFR (30,59) & UACR (30, 299) | 899 | 3.2 | 3.2 | 98.6 |
|  | eGFR (30, 59) & UACR ≥300 | 187 | 0.7 | 0.7 | 99.3 |
|  | eGFR <30 & UACR <30 | 85 | 0.3 | 0.3 | 99.6 |
|  | eGFR <30 & UACR (30, 299) | 75 | 0.3 | 0.3 | 99.8 |
|  | eGFR <30 & UACR ≥300 | 51 | 0.2 | 0.2 | 100.0 |
|  | Total | 28.344 | 100.0 | 100.0 |  |

eGFR, estimated Glomerular Filtration Rate (mL/min/1.73m^2^); UACR, Albumin to Creatinine Ratio (mg/g)
